# The Impact of Anthropomorphic Eco-Friendly Logos on Consumers’ Green Purchase Intention: A Moderated Mediation Model

**DOI:** 10.3390/bs16060965

**Published:** 2026-06-10

**Authors:** Yi An, Ji Xu, Dingbang Luh, Tiansheng Xia, Yibing Chen

**Affiliations:** School of Art and Design, Guangdong University of Technology, Guangzhou 510006, China

**Keywords:** anthropomorphism, perceived love, perceived hope, eco-friendly logos, environmental attitude, green purchase intention

## Abstract

Anthropomorphism is a widely used marketing strategy, yet less is known about how baby-schema anthropomorphic cues embedded in eco-friendly logos function as compact visual identity cues to promote consumers’ green purchase intention through positive emotional attribution. Drawing on baby-schema theory and mental-state attribution, we examine the impact of anthropomorphic eco-friendly logos on green purchase intention, the mediating roles of perceived love and perceived hope, their sequential pathway, and the moderating effect of environmental attitude. A within-subjects study was conducted with 299 valid participants in China, using established and adapted scale items for data collection. Our results demonstrated that anthropomorphic eco-friendly logos significantly enhanced green purchase intention. Perceived love and perceived hope each mediated this relationship, and the sequential pathway from perceived love to perceived hope was also significant. Moreover, environmental attitude positively moderated the link between anthropomorphic logos and perceived love, with a stronger effect among consumers with higher pro-environmental attitudes. These findings highlight a positive emotional attribution mechanism through which anthropomorphic eco-friendly logo cues promote green consumption and clarify the boundary role of environmental attitude.

## 1. Introduction

### 1.1. Eco-Friendly Logos and Anthropomorphic Design

Environmental protection has become a global priority, with more consumers recognizing the positive impact of eco-friendly products on sustainability. Eco-friendly products are defined as goods that significantly reduce environmental harm during their production, use, and disposal (Gao & Mattila, 2016). As consumers’ environmental awareness rises, environmental attributes play an increasingly critical role in their product selection (Shabbir et al., 2020). To identify the factors influencing green purchase intention, researchers have systematically investigated multiple dimensions, including information appeals (D. Yang et al., 2015), product presentation formats (H. Wang et al., 2020), individual and social factors (Mazar & Zhong, 2010), and product logo design (H. Li et al., 2023). Among these factors, the design is particularly relevant because logos serve as concise visual cues through which consumers recognize and evaluate green products. For instance, the authors of the existing studies have explored the effects of logo features such as color (Sundar & Kellaris, 2017), simplicity (Bossel et al., 2019), interstitial space (Gupta & Hagtvedt, 2021), letter case (Teng et al., 2021), cuteness (Septianto & Paramita, 2021), and anthropomorphic design (Laksmidewi et al., 2017) on consumer preferences. However, there has been less research on how baby-schema anthropomorphic cues embedded in eco-friendly logos, as compact and repeatedly encountered green identity cues, shape consumers’ green purchase intention through positive emotional and mental-state attributions.

Anthropomorphism refers to the tendency to attribute humanlike characteristics, intentions, emotions, or mental states to non-human agents (Epley et al., 2007). In consumer behavior, anthropomorphic design can make products, brands, and visual symbols appear more socially meaningful and emotionally engaging. The results of prior studies have demonstrated that anthropomorphic cues can enhance consumers’ responses to green products by increasing trust, perceived product effectiveness, recycling intention, and favorable brand attitudes (Laksmidewi et al., 2017; Lu et al., 2021; T. Wang et al., 2017; Septianto & Paramita, 2021). Baby-schema theory provides a useful explanation for why anthropomorphic eco-friendly logos may elicit perceived love. Because logos are static and compact visual identity cues, we focus on visual anthropomorphism rather than voice-based, role-based, or narrative forms of anthropomorphism that are more common in advertising, service encounters, or interactive brand communication. Infantile visual features, such as large eyes, round faces, and softened contours, can be directly embedded in logo design and can elicit caregiving motivation, empathic concern, and positive affective reactions (Glocker et al., 2009; Borgi et al., 2014). The results of related emotion research suggest that cuteness may evoke tender and affectionate responses, including kama muta, a feeling of being moved by love (Steinnes et al., 2019). Accordingly, we examine perceived love as a mental-state attribution to non-human visual agents rather than as interpersonal love, general brand affect, or long-term brand love. Eco-friendly logos differ from broader anthropomorphic stimuli because they appear directly on products and brand communications and serve as concise cues of environmental positioning. In this context, even subtle infantile features may influence how consumers interpret the product’s environmental value and social meaning.

In this study, we therefore focus on baby-schema anthropomorphic elements embedded in eco-friendly logos. In green consumption, such care-based affect may be particularly relevant because purchasing eco-friendly products involves concern for environmental protection, future generations, and collective well-being (Cavanaugh et al., 2015; Winterich & Haws, 2011). By transforming abstract environmental information into a more socially meaningful and emotionally engaging visual cue, anthropomorphic eco-friendly logos may increase consumers’ green purchase intention. Accordingly, we propose Hypothesis 1:

**H1.**  *Compared with conventional non-anthropomorphic eco-friendly logos, anthropomorphic eco-friendly logos increase consumers’ green purchase intention*.

### 1.2. Perceived Love and Perceived Hope

In this study, perceived love and perceived hope refer to consumers’ attribution of love-related and hope-related mental states to anthropomorphic eco-friendly logos, rather than to enduring brand love, general brand affect, or consumers’ own dispositional hope. Although love and hope may carry Christian or Western philosophical connotations in some streams of literature, the present study follows the perspective of mental-state attribution and consumer response research. Specifically, perceived love captures care-oriented warmth, nurturance, protective affection, and emotional closeness attributed to baby-schema anthropomorphic logo cues, whereas perceived hope captures a future-oriented positive expectation regarding environmental improvement, collective well-being, and a more sustainable future. In the Chinese and broader Asian cultural context, these responses can be understood as care-oriented affection and future-oriented positive expectation rather than as religious meanings of love and hope.

Perceived love is introduced as the first mediator because baby-schema anthropomorphic features are closely associated with caregiving motivation, empathic concern, and affectionate responses (Glocker et al., 2009; Borgi et al., 2014). Compared with general positive affect or brand warmth, perceived love more directly captures the care-oriented attribution elicited by infantile anthropomorphic cues. Because green consumption often involves concern for environmental protection, future generations, and collective well-being (Cavanaugh et al., 2015), such care-based attribution may increase consumers’ willingness to choose eco-friendly products. Accordingly, we propose Hypothesis 2:

**H2.**  *Perceived love mediates the positive effect of anthropomorphic eco-friendly logos on green purchase intention*.

Perceived hope is introduced as a second mediator because green purchase intention is inherently future-oriented and involves expectations about environmental improvement and benefits for future generations. In this study, perceived hope does not refer to consumers’ dispositional hope; rather, it captures the extent to which consumers attribute a hope-related mental state to the anthropomorphic eco-friendly logo. Hope has been conceptualized as an anticipatory emotion associated with a pleasurable expectation of a desired future event (Winterich & Haws, 2011). In the environmental domain, hope is closely related to expectations of environmental improvement and concern for the living conditions of future generations (Tong et al., 2021). Lin and Fung (2026) found that although older adults did not perceive higher ecological risks than younger adults, they reported more pro-environmental behaviors, suggesting the importance of concern for future generations. In consumption settings, hope can enhance positive attitudes toward products, promote favorable expectations about future outcomes, and shape purchase decisions (MacInnis & De Mello, 2005; Tong et al., 2021). Therefore, anthropomorphic eco-friendly logos may elicit perceived hope by leading consumers to associate the logo with the possibility of positive environmental change. Accordingly, we propose Hypothesis 3:

**H3.**  *Perceived hope mediates the positive effect of anthropomorphic eco-friendly logos on green purchase intention*.

The proposed perceived love and perceived hope sequence is specific to the context of anthropomorphic eco-friendly logos and does not imply that hope can only arise after love. Anthropomorphic eco-friendly logos may elicit perceived hope directly by leading consumers to associate the logo with the possibility of positive environmental change. Simultaneously, infantile anthropomorphic cues may first lead consumers to perceive the logo as lovable, caring, and socially meaningful. In green consumption, this immediate care-based attribution is important because sustainable choices often involve concern beyond immediate self-interest, including concern for others, future generations, and collective environmental well-being (Cavanaugh et al., 2015; Winterich & Haws, 2011). Once perceived love is elicited, consumers may become more receptive to the future-oriented environmental meaning of the logo, thereby strengthening perceived hope. Thus, anthropomorphic eco-friendly logos may influence green purchase intention through both an independent hope-related pathway and a sequential pathway from perceived love to perceived hope. Accordingly, we propose Hypothesis 4:

**H4.**  *Perceived love and perceived hope sequentially mediate the effect of anthropomorphic eco-friendly logos on green purchase intention*.

### 1.3. Environmental Attitude

There are significant individual differences in the impact of anthropomorphic design on consumers’ affective perceptions, and environmental attitude—an individual’s core evaluative tendency toward the natural environment (Sockhill et al., 2022)—may play a key moderating role in this process. Previous research has established that environmental attitude is a critical antecedent of pro-environmental behavior: individuals with strong pro-environmental attitudes are not only more likely to form positive attitudes toward environmental protection (Karimi et al., 2022; Pirchio et al., 2021), but also to drive pro-environmental behavior by strengthening behavioral intention (Zarei et al., 2020).

Consumers with stronger pro-environmental attitudes are more likely to form favorable judgments of eco-friendly products (Sörqvist et al., 2015; Wiedmann et al., 2014) and to experience emotional satisfaction from pro-environmental behavior. The moderating role of environmental attitude can be further explained by the inclusion of nature in self-perspective. Individuals with strong pro-environmental attitudes are more likely to incorporate nature into their self-concept, making environmental cues psychologically closer, more self-relevant, and more value-congruent (Schultz, 2002; Whitmarsh & O’Neill, 2010). When exposed to baby-schema anthropomorphic eco-friendly logos, these consumers may therefore interpret the logo’s cuteness and humanlike features as meaningful care-related environmental cues rather than as merely decorative design elements. This heightened self-relevance and value congruence may strengthen the effect of anthropomorphic logos on perceived love. In contrast, consumers with weaker pro-environmental attitudes may be less likely to connect anthropomorphic logo cues with environmental care, resulting in a weaker affective response. Based on the above analysis, we propose Hypothesis 5:

**H5.**  *Environmental attitude positively moderates the relationship between anthropomorphic eco-friendly logos and perceived love, such that anthropomorphic logos elicit stronger perceived love among consumers with higher pro-environmental attitudes*.

### 1.4. Research Gaps and Contributions

The results of previous studies have demonstrated that anthropomorphism can encourage pro-environmental responses, often through cognitive mechanisms such as connectedness to nature and perceived responsibility, or through negative emotional mechanisms such as environmental guilt and anxiety (Tam, 2019; Y. Yang et al., 2023). However, these perspectives do not fully explain how baby-schema anthropomorphic cues embedded in eco-friendly logos generate positive emotional attribution and future-oriented motivation in consumer decision-making. Although the authors of prior studies have examined logo features such as color, simplicity, interstitial space, letter case, and cuteness (Gupta & Hagtvedt, 2021; Teng et al., 2021; Septianto & Paramita, 2021), the positive emotional pathway linking anthropomorphic eco-friendly logo cues to green purchase intention remains insufficiently clarified. This omission is important because logos are condensed and repeatedly encountered identity cues that help consumers recognize a product’s environmental positioning. Anthropomorphic elements may therefore shape not only visual appeal but also consumers’ social and emotional interpretation of green products.

In the present study, we address these gaps using three approaches. First, we shift the focus of anthropomorphism research from broad product presentation, advertising characters, and nature anthropomorphism to eco-friendly logos as specific visual identity cues. Second, we develop a positive emotional attribution framework in which perceived love captures the immediate care-based response to infantile anthropomorphic cues, perceived hope reflects future-oriented expectations about environmental improvement, and the love–hope sequence explains how care-based affect may be transformed into future-oriented green purchase motivation. Third, we examine environmental attitude as a boundary condition that determines when anthropomorphic eco-friendly logos are more likely to elicit perceived love and enhance green purchase intention.

## 2. Materials and Methods

### 2.1. Participants

This study was conducted in China using Wenjuanxing, an online questionnaire platform. Participants were recruited through convenience sampling and completed the questionnaire anonymously. Convenience sampling was used as a participant recruitment strategy because no complete sampling frame of green consumers was available. This sampling strategy mainly concerns external validity and population-level generalizability. By contrast, the internal validity of the experimental test was addressed through the within-subjects manipulation, multiple logo stimuli for each logo type, and counterbalanced presentation order.

The sample was not restricted to Guangzhou, although it was regionally concentrated in Guangdong Province, where the authors’ institution is located. Based on the available regional information, approximately 83% of the valid respondents were from Guangdong Province, while the remaining participants came from other provinces or municipalities in China, including Beijing, Shanghai, Jiangsu, Zhejiang, and Sichuan. A total of 318 questionnaires were distributed, and 299 valid questionnaires were collected, yielding an effective response rate of 94%. The sample included 139 male and 160 female participants, aged between 18 and 35 years, with a mean age of 22.34 years (SD = 2.61). All participants provided informed consent electronically before completing the questionnaire and received a small monetary reward after participation. This study was approved by the Academic Ethics Committee of Guangdong University of Technology (Approval No.: GDUTXS2024127).

### 2.2. Power Analysis

We adopted a Monte Carlo power analysis designed for indirect effects (Schoemann et al., 2017) to determine the minimum required sample size. Small-to-moderate correlations between variables were assumed in the model. The following parameters were specified: statistical power = 0.80, confidence level = 99%, 10,000 Monte Carlo replications, and 20,000 bootstrap draws per replication. The power analysis indicated that the required sample size for this research setting should be at least 287. Because the present study obtained 299 valid responses, the sample size exceeded the minimum requirement and provided sufficient statistical power for testing the proposed mediation and moderated mediation model.

### 2.3. Research Design and Stimuli

We adopted a single-factor, two-level within-subjects design. The within-subjects factor was logo type, including anthropomorphic eco-friendly logos and conventional non-anthropomorphic eco-friendly logos. Because all participants evaluated both logo types, the study did not require random assignment to separate anthropomorphic and non-anthropomorphic conditions. Instead, the key experimental control was to reduce potential order effects and stimulus-specific bias. To reduce order effects, the presentation order of the two logo sets was fully counterbalanced across participants: approximately half of the participants viewed the anthropomorphic logo set first and then the non-anthropomorphic logo set, whereas the other half viewed the two sets in the opposite order.

**Figure 1:**
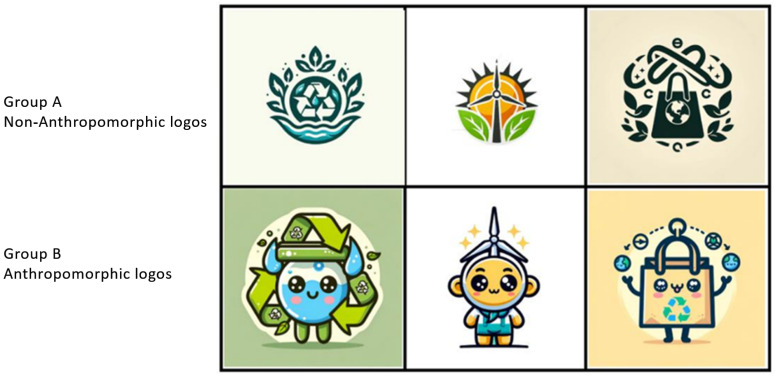
Experimental stimuli. Group A = non-anthropomorphic logos; Group B = anthropomorphic logos.

To reduce stimulus-specific bias, multiple logo stimuli were developed for each logo type. Specifically, three anthropomorphic eco-friendly logos and three conventional non-anthropomorphic eco-friendly logos were included, yielding six stimuli in total (see Figure 1). The anthropomorphic logos incorporated infantile humanlike features, such as large eyes, round faces, and softened contours, whereas the conventional non-anthropomorphic logos included standard environmental symbols, such as recycling signs, leaves, and earth-related icons, without humanlike facial or bodily features. All logos were presented in a standardized digital format with comparable size, resolution, and visual complexity. Participants completed the corresponding evaluations after exposure to each logo set.

A pretest was conducted with 42 participants who did not take part in the formal experiment. These participants rated the two stimulus sets on anthropomorphism, cuteness, visual complexity, processing fluency, visual novelty, and familiarity. The results highlighted significant differences in anthropomorphism (M_A_ = 3.98, M_N_ = 2.54, t(41) = 6.53, *p* < 0.001) and cuteness scores (M_A_ = 4.35, M_N_ = 3.39, t(41) = 4.61, *p* < 0.001) between the two sets of materials; in comparison, no significant differences were observed in visual complexity (M_A_ = 3.01, M_N_ = 3.17, t(41) = −0.92, *p* = 0.359), processing fluency (M_A_ = 4.15, M_N_ = 4.11, t(41) = 0.34, *p* = 0.737), visual novelty (M_A_ = 3.94, M_N_ = 3.98, t(41) = −0.26, *p* = 0.797), and familiarity (M_A_ = 3.33, M_N_ = 3.40, t(41) = −0.35, *p* = 0.727).

### 2.4. Measures

#### 2.4.1. Green Purchase Intention (GPI)

Green purchase intention refers to consumers’ subjective tendency to purchase a product after becoming aware of its green attributes (Oliver & Lee, 2010). In this study, we adopted the 3-item scale developed by R. Y. K. Chan (2001) to measure the green purchase intention of Chinese consumers. All items were rated on a 7-point Likert scale, where 1 = “very unwilling to buy” and 7 = “very willing to buy”. Higher scores indicated stronger green purchase intention. The Cronbach’s α of this scale in the current study was 0.906.

#### 2.4.2. Revised New Ecological Paradigm Scale (NEP-R)

The NEP-R Scale is a widely used instrument for measuring individuals’ fundamental beliefs about the human–nature relationship, which shape environmental attitudes and pro-environmental behavior (Dorward et al., 2024). We used the revised 15-item NEP-R Scale developed by Dunlap et al. (2000), a widely used version of the scale. Items were rated on a 5-point Likert scale. Odd-numbered items measured pro-environmental attitudes, while even-numbered items were reverse-coded. After reverse coding, higher total scores indicated stronger pro-environmental attitudes and firmer pro-environmental beliefs. The Cronbach’s α of this scale in the current study was 0.751.

#### 2.4.3. Attribution of Mental States (AMS-Q)

Anthropomorphism involves projecting human traits and mental states onto non-human agents, which can facilitate interaction with these targets (Ding et al., 2021). We employed the Attribution of Mental States Questionnaire (AMS-Q) to measure participants’ perceived hope and perceived love toward anthropomorphic eco-friendly logos. The AMS-Q is appropriate for this purpose because it assesses the extent to which individuals attribute psychological states to non-human objects.

The AMS-Q is a standardized and validated scale, primarily developed to evaluate the extent to which individuals attribute mental, emotional, intentional, perceptual, and cognitive states to a specific target (Miraglia et al., 2023). It has been widely used in studies on non-human agents across different age groups, including children (Di Dio et al., 2020; Manzi et al., 2020) and adults (Manzi et al., 2021; Di Dio et al., 2025), with research subjects covering various non-human entities such as robots and virtual avatars. The AMS-Q was selected because the stimuli in this study were non-human visual agents, namely logos, rather than established brands or interpersonal partners.

We selected one love-related item and one hope-related item from the AMS-Q as study-specific single-item indicators. Perceived love was measured using the item “It has the capacity to love,” and perceived hope was measured using the item “It can make a wish.” Both items were rated on a 5-point Likert scale. The full scale items used in this study are presented in Appendix A.

### 2.5. Common Method Bias Test

Harman’s single-factor test was conducted to examine potential common method bias (Zhou & Long, 2004). The exploratory factor analysis extracted 10 factors with eigenvalues greater than 1. The first unrotated factor accounted for 23.57% of the total variance, which was below the commonly used threshold of 40%. These results suggest that common method bias was unlikely to seriously affect the findings of this study.

## 3. Results

### 3.1. Descriptive Statistics and Correlation Analysis

Pearson correlation analysis was conducted to examine the associations between logo type, perceived love, perceived hope, green purchase intention, and environmental attitude. Descriptive statistics and the correlation matrix for all variables are presented in Table 1. Logo type was significantly and positively correlated with perceived love; perceived love was significantly and positively correlated with perceived hope; and logo type, perceived love, and perceived hope were all significantly and positively correlated with green purchase intention.

### 3.2. Mediation Effect Test

The sequential mediation effect was tested using Model 6 of the SPSS 26.0 PROCESS macro with 5000 bias-corrected bootstrap samples (Hayes, 2017). The independent variable, dependent variable, and mediating variables were entered into the model, and 95% confidence intervals (CIs) were calculated.

**Figure 2:**
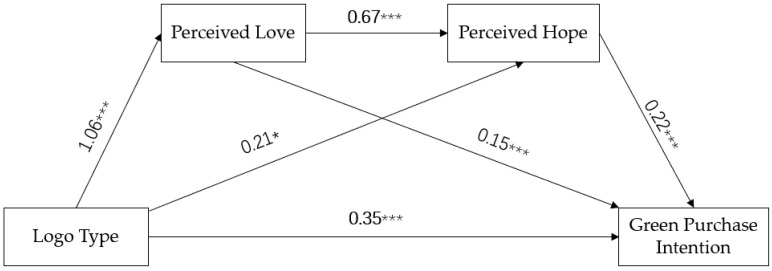
Sequential mediation model of the effect of logo type on green purchase intention. Note: * *p* < 0.05, *** *p* < 0.001.

The regression results (Table 2) showed that logo type significantly and positively predicted perceived love (*b* = 1.06, *p* < 0.001), perceived hope (*b* = 0.21, *p* = 0.018), and green purchase intention (*b* = 0.35, *p* < 0.01). Perceived love significantly and positively predicted both perceived hope (*b* = 0.67, *p* < 0.001) and green purchase intention (*b* = 0.15, *p* < 0.001). Perceived hope also significantly and positively predicted green purchase intention (*b* = 0.22, *p* < 0.001). The mediation model is illustrated in Figure 2.

The mediation effect analysis (Table 3) distinguished the direct effect from the indirect effects. After perceived love and perceived hope were included in the model, the direct effect of logo type on green purchase intention remained significant, indicating partial mediation. The total indirect effect was significant (effect = 0.36, 95% CI [0.26, 0.47]). Among the three indirect pathways, the indirect effect through perceived love was significant (effect = 0.16, 95% CI [0.09, 0.25]), and the independent indirect effect through perceived hope was also significant (effect = 0.04, 95% CI [0.01, 0.09]). In addition, the sequential indirect effect through perceived love and then perceived hope was significant (effect = 0.15, 95% CI [0.10, 0.22]). Therefore, H2, H3, and H4 were supported. These results indicate that anthropomorphic eco-friendly logos influence green purchase intention through both independent emotional attribution pathways and a sequential pathway from perceived love to perceived hope. Indirect effects were considered significant when their 95% bias-corrected confidence intervals did not include zero.

### 3.3. Moderated Mediation Effect Test

To test the moderating effect of environmental attitude, Model 83 of the SPSS PROCESS macro was used to examine whether environmental attitude moderated the indirect effect of logo type on green purchase intention via perceived love. The results demonstrated that environmental attitude had a significant moderating effect on the relationship between logo type and perceived love (95% CI [0.1853, 1.3099], excluding 0), as shown in Table 4.

Specifically, for individuals with low pro-environmental attitudes (1 SD below the mean), logo type had a significant positive effect on perceived love (*b* = 0.80, *t* = 5.57, *p* < 0.001). For individuals with high pro-environmental attitudes (1 SD above the mean), the positive effect of logo type on perceived love was even stronger and more significant (*b* = 1.32, *t* = 9.26, *p* < 0.001). These findings indicate that environmental attitude amplifies the positive effect of anthropomorphic logos on perceived love.

**Figure 3:**
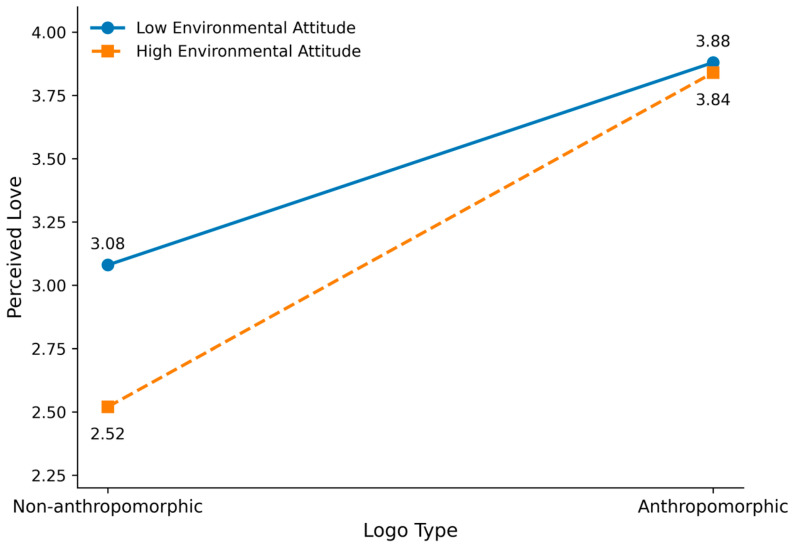
Moderating effect of environmental attitude on the relationship between logo type and perceived love.

The results of conditional indirect effect analysis further confirmed that environmental attitude moderated the indirect effect of logo type on green purchase intention via perceived love, as presented in Table 5 and Figure 3. For individuals with low pro-environmental attitudes (1 SD below the mean), the indirect effect was significant (effect = 0.1143, SE = 0.0290, 95% CI [0.0619, 0.1749]). For individuals with high pro-environmental attitudes (1 SD above the mean), the indirect effect was stronger and also significant (effect = 0.1901, SE = 0.0408, 95% CI [0.1184, 0.2789]). The index of moderated mediation was significant because its 95% confidence interval did not include zero. Thus, H5 was supported.

To provide a clearer overview of the empirical results, the testing outcomes of the five hypotheses are summarized in Table 6. Overall, H1, H2, H3, H4, and H5 were supported. These results indicate that anthropomorphic eco-friendly logos directly enhanced green purchase intention and indirectly influenced green purchase intention through perceived love, perceived hope, and the sequential pathway from perceived love to perceived hope.

## 4. Discussion

### 4.1. Green Consumption and Purchase Intention

Our findings demonstrate that anthropomorphic eco-friendly logos can enhance green purchase intention by activating positive emotional attributions, including perceived love and perceived hope. Our results extend prior research on logo design, primarily focused on examining visual features such as color, spacing, letter case, and cuteness, by showing that anthropomorphic elements can also shape consumers’ responses to green products through care-based and future-oriented emotional mechanisms. Consistent with the existing environmentalism research, studies have demonstrated that anthropomorphizing nature can promote green behavior. For example, E. Y. Chan (2021) showed that anthropomorphizing water as “Mr. Water” promoted water conservation. Similarly, the present findings suggest that anthropomorphic cues can promote green responses even when they are embedded in compact logo designs rather than in broader environmental messages. These findings complement rather than duplicate prior research on anthropomorphism and pro-environmental behavior. For example, Y. Yang et al. (2023) demonstrated that anthropomorphizing nature can promote pro-environmental behavior through connectedness to nature and environmental guilt. The present study differs in that we focus on anthropomorphic eco-friendly logos as product-level visual identity cues and identify positive emotional attribution pathways involving perceived love, perceived hope, and the sequential process from perceived love to perceived hope. This distinction suggests that anthropomorphism may influence green outcomes through different psychological routes depending on whether the anthropomorphized object is nature itself or a concrete brand-related visual symbol.

### 4.2. The Mediating Roles of Perceived Love and Perceived Hope

Using AMS-Q items that capture mental-state attribution toward non-human agents, we examined consumers’ psychological responses to anthropomorphic versus conventional eco-friendly logos. The results demonstrated that anthropomorphic eco-friendly logos with baby-schema features elicited both perceived love and perceived hope, which in turn enhanced green purchase intention. In addition, the sequential pathway from perceived love to perceived hope was significant. These findings indicate that anthropomorphic eco-friendly logos can influence green purchase intention through both independent positive emotional attribution pathways and a sequential pathway in which care-based attribution strengthens future-oriented hope.

The mediation results can be understood within the specific context of green consumption. Perceived love reflects an immediate care-based attribution elicited by infantile anthropomorphic cues, whereas perceived hope reflects future-oriented expectations regarding environmental improvement and collective well-being. Consistent with research on positive emotions and sustainable consumption (Cavanaugh et al., 2015; Winterich & Haws, 2011) and hope in consumer decision-making (MacInnis & De Mello, 2005; Tong et al., 2021), anthropomorphic eco-friendly logos may directly elicit hope-related attribution by linking the product with a desirable environmental future. Simultaneously, the significant sequential pathway suggests that a care-based response toward the anthropomorphic logo can further strengthen consumers’ future-oriented interpretation of the product’s environmental value.

The present findings are consistent with prior research demonstrating that anthropomorphic design can enhance consumers’ favorable responses by increasing emotional resonance and humanlike perception (Epley et al., 2007; Laksmidewi et al., 2017; X. Wang et al., 2020). They also align with baby-schema theory, which suggests that infantile features can evoke caregiving motivation and empathic concern (Glocker et al., 2009; Borgi et al., 2014).

### 4.3. The Moderating Role of Environmental Attitude

The moderating effect of environmental attitude suggests that anthropomorphic logo cues are not interpreted uniformly by all consumers. Consumers with stronger pro-environmental attitudes may perceive environmental objects as more self-relevant and emotionally meaningful, making them more responsive to baby-schema anthropomorphic cues. As a result, anthropomorphic eco-friendly logos elicited stronger perceived love among consumers with higher environmental attitudes.

In the present study, we focused on baby-schema visual anthropomorphism, which is likely to elicit care-related responses. However, different forms of anthropomorphic logo design may elicit different psychological mechanisms. For example, adult-like, animal-like, or competence-oriented anthropomorphic logos may influence consumers through perceived responsibility, trust, competence, or moral concern rather than perceived love. Future research could compare different anthropomorphic styles to examine whether the moderating role of environmental attitude remains stable across alternative logo designs. The moderating effect of environmental attitude also has important implications for targeting strategies. Because the effect of anthropomorphic eco-friendly logos was stronger among consumers with higher environmental attitudes, such logos may be particularly effective in reinforcing existing pro-environmental values and strengthening emotional engagement among consumers who already value sustainability. For consumers with weaker environmental attitudes, cute anthropomorphic cues alone may be insufficient to generate strong care-based responses. In addition, when consumers are skeptical of green claims, anthropomorphic logo design should be combined with credible environmental information, certification cues, and concrete evidence of product sustainability. The results of previous studies demonstrate that green skepticism can reduce favorable responses to green marketing and purchase intentions, while greenwashing can undermine green trust through consumer confusion and perceived risk (Leonidou & Skarmeas, 2017; Chen & Chang, 2013).

### 4.4. Limitations and Future Research Directions

This study has several limitations that should be addressed in future research.

First, the sample was relatively young and regionally concentrated, with participants aged between 18 and 35 years and approximately 83% of valid respondents from Guangdong Province. Because the study relied on an online convenience sample, the findings may not fully generalize to older consumers, non-student populations, consumers from other regions, or non-Chinese cultural contexts. In addition, although perceived love and perceived hope are treated in this study as secular affective and mental-state attribution constructs, their semantic meanings and emotional associations may vary across cultural contexts. The authors of future studies should replicate the model with more diverse and cross-cultural samples to examine whether consumers from different cultural backgrounds interpret care-oriented affection and future-oriented hope in similar or different ways when responding to anthropomorphic eco-friendly logos. Future research should also incorporate behavioral choice tasks, willingness-to-pay measures, or field data to improve external validity.

Second, we employed a questionnaire-based stimulus design, which captures consumers’ immediate responses to logo exposure but does not allow us to determine whether perceived love and perceived hope lead to repeated green purchases over time. Although multiple logo stimuli were used for each logo type and the presentation order was counterbalanced to reduce stimulus-specific and order-related bias, these procedures cannot substitute for probability-based sampling, stricter randomized order assignment where feasible, longitudinal designs, or field-based evidence.

The authors of future studies could adopt longitudinal panel designs to examine whether perceived love and perceived hope measured after exposure to anthropomorphic eco-friendly logos predict subsequent green purchase behavior, repeated purchases, or brand loyalty over time. In addition, eye-tracking, behavioral choice tasks, willingness-to-pay measures, and field experiments using real or professionally designed eco-friendly logos could provide stronger evidence for the psychological and behavioral effects of anthropomorphic logo design.

Third, perceived love and perceived hope were measured using single AMS-Q items that capture mental-state attribution toward non-human agents. The authors of future studies could use dedicated measures of state hope, brand love, perceived emotional warmth, or kama muta to examine whether the love→hope sequence remains stable across alternative measurement instruments. In addition, because the data were collected through self-report questionnaires, the authors of such studies could use multi-source data, behavioral records, or time-lagged designs to reduce common method concerns.

Lastly, we asked participants to evaluate logo stimuli directly, without embedding the logos in specific product contexts. The effectiveness of cute anthropomorphic eco-friendly logos may vary across product types and levels of product involvement. The results of previous studies have demonstrated that consumers with different involvement levels allocate visual attention and process product information differently (Behe et al., 2015). Recent ELM-based consumer research findings also suggest that argument quality functions as a central-route cue, whereas design and social elements can operate as peripheral-route cues (Cyr et al., 2018). In green consumption, product involvement and attribute importance may further shape consumers’ purchase intentions (Mark et al., 2023). Therefore, cute anthropomorphic logos may be more effective for low-involvement eco-friendly products, such as recycled paper or daily consumables, where visual and affective cues play a relatively stronger role. For high-involvement products, such as electric vehicles, consumers may rely more heavily on product performance, price, safety, environmental certification, and functional reliability. The intensity of cuteness may also represent a boundary condition. The results of previous studies suggest that cuteness tends to enhance perceived brand warmth rather than brand competence, whereas competence-related impressions may be especially important for products requiring reliability, safety, or technological performance (B. Li et al., 2022; Lu et al., 2021). The authors of future studies should compare different product categories, product-involvement levels, and degrees of anthropomorphic cuteness to test the boundary conditions of anthropomorphic eco-friendly logo design.

## 5. Conclusions

In this study, we examined how anthropomorphic eco-friendly logos influence consumers’ green purchase intention through perceived love and perceived hope and how this process depends on environmental attitude. H1, H2, H3, H4, and H5 were supported by the results. Specifically, anthropomorphic eco-friendly logos significantly increased green purchase intention. Perceived love and perceived hope each served as significant mediators, and the sequential pathway from perceived love to perceived hope was also significant. In addition, environmental attitude strengthened the effect of anthropomorphic logos on perceived love, which can be explained by the greater self-nature overlap, self-relevance, and value congruence among consumers with stronger pro-environmental attitudes.

The indirect effect through perceived love was the largest among the three indirect pathways, suggesting that care-based attribution plays an immediate and important role in shaping green purchase intention. However, perceived hope also served as a significant independent mediator, indicating that anthropomorphic eco-friendly logos can directly evoke future-oriented expectations about positive environmental change. The significant sequential pathway further suggests that perceived love can strengthen perceived hope, transforming an immediate care-based response into future-oriented green purchase motivation.

Practically, eco-friendly brands may incorporate moderate anthropomorphic cues into logo design to strengthen consumers’ affective responses and green purchase intention. However, cuteness should be balanced with competence and credibility, especially for high-involvement products for which consumers rely heavily on product performance, safety, certification, and functional reliability. For consumers who are less environmentally engaged or skeptical of green claims, anthropomorphic logo cues should be supported by verifiable sustainability information, such as eco-labels, certification sources, and specific environmental claims, because credible and specific green information can reduce skepticism and strengthen consumer trust (Atkinson & Rosenthal, 2014; Schmuck et al., 2018). Communication strategies may further highlight both the care-related meaning of the anthropomorphic logo and the product’s contribution to environmental improvement and future generations, thereby strengthening perceived love and perceived hope simultaneously.

## Figures and Tables

**Table 1:** Means, standard deviations, and correlation matrix for all variables (*n* = 299).

	M	SD	1	2	3	4	5
1. Logo Type	-	-	-				
2. Perceived Love	3.33	1.35	0.39 **	-			
3. Perceived Hope	3.35	1.36	0.34 **	0.69 **	-	-	
4. Environmental Attitude	4.27	0.35	0.03	−0.11 *	−0.05	-	
5. Green Purchase Intention	4.79	1.02	0.35 **	0.47 **	0.49 **	0.12 **	-

Note: * *p* < 0.05, ** *p* < 0.01.

**Table 2:** Regression results for the mediating roles of perceived love and perceived hope between logo type and green purchase intention.

	Perceived Love			Perceived Hope			Green Purchase Intention		
	*B*	*SE*	*t*	*B*	*SE*	*t*	*B*	*SE*	*t*
Constant	1.74	0.16	10.78 ***	0.82	0.14	5.89 ***	3.04	0.13	24.23 ***
Logo Type	1.06	0.10	10.37 ***	0.21	0.09	2.38 *	0.35	0.08	4.53 ***
Perceived Love				0.67	0.03	20.69 ***	0.15	0.04	4.15 ***
Perceived Hope							0.22	0.04	5.99 ***
*R* ^2^	0.15			0.48			0.29		
*F*	107.61			278.96			82.58		

Note: * *p* < 0.05, *** *p* < 0.001.

**Table 3:** Bootstrap 95% confidence intervals for mediation effect pathways.

	Effect	Boot SE	Boot LLCI	Boot ULCI	Relative Mediation Effect
TOTAL	0.36	0.05	0.26	0.47	
Ind1: Logo type→Perceived love→ Green purchase intention	0.16	0.04	0.09	0.25	45.31%
Ind2: Logo type→Perceived hope→ Green purchase intention	0.04	0.02	0.01	0.09	12.44%
Ind3: Logo type→Perceived love→ Perceived hope→Green purchase intention	0.15	0.03	0.10	0.22	42.25%

Note: Number of bootstrap samples = 5000. Indirect effects were considered significant when the 95% bias-corrected confidence interval did not include zero. Regression coefficients were evaluated using two-tailed tests.

**Table 4:** Moderating effect of environmental attitude on the mediation pathway via perceived love.

	Perceived Love			Perceived Hope			Green Purchase Intention		
	*B*	*SE*	*t*	*B*	*SE*	*t*	*B*	*SE*	*t*
Constant	3.33	0.05	65.95 ***	1.13	0.11	9.86 ***	3.56	0.11	32.83 ***
Logo Type	1.06	0.10	10.49 ***	0.21	0.09	2.38 *	0.35	0.08	4.53 ***
Perceived Love				0.67	0.03	20.70 ***	0.15	0.04	4.15 ***
Perceived Hope							0.22	0.04	5.99 ***
Environmental Attitude	−0.42	0.14	−2.94 **						
Logo Type × Environmental Attitude	0.75	0.29	2.61 **						
*R* ^2^	0.17			0.48			0.29		
*F*	41.84			278.96			80.58		

Note: * *p* < 0.05, ** *p* < 0.01, *** *p* < 0.001.

**Table 5:** Bootstrap results for the conditional indirect effect of environmental attitude.

Moderator	Boot Indirect Effect	Boot SE	BC 95% CI	
			Lower	Upper
−1 SD	0.1143	0.0290	0.0619	0.1749
+1 SD	0.1901	0.0408	0.1184	0.2789

Note: Number of bootstrap samples = 5000. SE = standard error. BC CI = bias-corrected confidence interval.

**Table 6:** Summary of hypothesis testing results.

Hypothesis	Hypothesized Relationship	Result
H1	Anthropomorphic design of eco-friendly logos increases consumers’ green purchase intention.	Supported
H2	Perceived love mediates the positive effect of anthropomorphic eco-friendly logos on green purchase intention.	Supported
H3	Perceived hope mediates the positive effect of anthropomorphic eco-friendly logos on green purchase intention.	Supported
H4	Perceived love and perceived hope sequentially mediate the effect of anthropomorphic eco-friendly logos on green purchase intention.	Supported
H5	Environmental attitude positively moderates the relationship between anthropomorphic eco-friendly logos and perceived love.	Supported

Note: “Supported” indicates that the hypothesized effect was statistically significant or that the bootstrap confidence interval did not include zero.

## Data Availability

The data presented in this study are available on request from the corresponding author.

## Abbreviations

The following abbreviations are used in this manuscript:

GPI	Green Purchase Intention
NEP-R	Revised New Ecological Paradigm Scale
AMS-Q	Attribution of Mental States Questionnaire
SD	Standard Deviation
SE	Standard Error
CI	Confidence Interval
LLCI	Lower-Limit Confidence Interval
ULCI	Upper-Limit Confidence Interval

## Appendix A

**Table A1:** Full items of scales adopted in this study.

Questionnaire Name	Scale Items	Reference
GPI	I am willing to buy this environmentally friendly product to conserve energy. I will purchase this environmentally friendly product for the sake of environmental protection. The possibility of me buying this environmentally friendly product is very high.	R. Y. K. Chan (2001)
NEP-R	We are approaching the limit of the number of people the earth can support. Humans have the right to modify the natural environment to suit their needs. When humans interfere with nature, it often produces disastrous consequences. Human ingenuity will ensure that we do not make the earth uninhabitable. Humans are severely abusing the environment. The earth has plenty of natural resources if we just learn how to develop them. Plants and animals have as much right as humans to exist. The balance of nature is strong enough to cope with the impacts of modern industrial nations. Despite our special abilities, humans are still subject to the laws of nature. The so-called “ecological crisis” facing humankind has been greatly exaggerated. The earth is like a spaceship with limited room and resources. Humans were meant to rule over the rest of nature. The balance of nature is very delicate and easily upset. Humans will eventually learn enough about how nature works to be able to control it. If things continue on their present course, we will soon experience a major ecological catastrophe.	Dunlap et al. (2000)
AMS-Q	It has the ability to learn autonomously. It has the ability to think independently. It has the ability to remember. It can make a decision on its own. It has the ability to understand. It can dream. It has the ability to imagine. It can have fun. It has the capacity to love. It can be happy. It can have the intention to do something. It may want to do something. It can make a wish.	Di Dio et al. (2025)

Note: GPI = Green Purchase Intention; NEP-R = Revised New Ecological Paradigm Scale; AMS-Q = Attribution of Mental States Questionnaire.
